# Extracellular Vesicle Release Promotes Viral Replication during Persistent HCV Infection

**DOI:** 10.3390/cells10050984

**Published:** 2021-04-22

**Authors:** Yucel Aydin, Ali Riza Koksal, Venu Reddy, Dong Lin, Hanadi Osman, Zahra Heidari, Sadeq Mutlab Rhadhi, William C Wimley, Mansour A Parsi, Srikanta Dash

**Affiliations:** 1 Department of Pathology and Laboratory Medicine, Tulane University Health Sciences Center, New Orleans, LA 70112, USA; yaydin@tulane.edu (Y.A.); vreddy2@tulane.edu (V.R.); dlin6@tulane.edu (D.L.); hosman1@tulane.edu (H.O.); srhadhi@tulane.edu (S.M.R.); 2 Department of Medicine, Division of Gastroenterology and Hepatology, Tulane University Health Sciences Center, New Orleans, LA 70112, USA; akoksal@tulane.edu (A.R.K.); mparsi@tulane.edu (M.A.P.); 3 Department of Chemical and Biomedical Engineering, Tulane University, New Orleans, LA 70112, USA; zheidari@tulane.edu; 4 Department of Biochemistry and Molecular Biology, Tulane University Health Sciences Center, New Orleans, LA 70112, USA; wwimley@tulane.edu; 5 Southeast Louisiana Veterans Health Care System, 2400 Canal Street, New Orleans, LA 70119, USA

**Keywords:** hepatitis C virus, HCV, endoplasmic reticulum stress, autophagy, multivesicular body, MVB, extracellular vesicles, EVs, exosomes, double-stranded RNA

## Abstract

Hepatitis C virus (HCV) infection promotes autophagic degradation of viral replicative intermediates for sustaining replication and spread. The excessive activation of autophagy can induce cell death and terminate infection without proper regulation. A prior publication from this laboratory showed that an adaptive cellular response to HCV microbial stress inhibits autophagy through beclin 1 degradation. The mechanisms of how secretory and degradative autophagy are regulated during persistent HCV infection is unknown. This study was performed to understand the mechanisms of viral persistence in the absence of degradative autophagy, which is essential for virus survival. Using HCV infection of a CD63-green fluorescence protein (CD63-GFP), labeled stable transfected Huh-7.5 cell, we found that autophagy induction at the early stage of HCV infection increased the degradation of CD63-GFP that favored virus replication. However, the late-stage of persistent HCV infection showed impaired autophagic degradation, leading to the accumulation of CD63-GFP. We found that impaired autophagic degradation promoted the release of extracellular vesicles and exosomes. The impact of blocking the release of extracellular vesicles (EVs) on virus survival was investigated in persistently infected cells and sub-genomic replicon cells. Our study illustrates that blocking EV and exosome release severely suppresses virus replication without effecting host cell viability. Furthermore, we found that blocking EV release triggers interferon lambda 1 secretion. These findings suggest that the release of EVs is an innate immune escape mechanism that promotes persistent HCV infection. We propose that inhibition of extracellular vesicle release can be explored as a potential antiviral strategy for the treatment of HCV and other emerging RNA viruses.

## 1. Introduction

The Hepatitis C virus (HCV) is a blood-borne pathogen causing chronic inflammation of the liver without any significant symptoms over several decades that, if untreated, leads to cirrhosis and, potentially, to the development of hepatocellular carcinoma (HCC) [1,2,3]. Approved direct-acting antivirals (DAAs) can cure most cases of chronic HCV infection and, if prescribed early enough, can prevent the progression of cirrhosis [4]. An HCV cure reduces liver inflammation, the progression of liver fibrosis, and HCC development, which decreases HCV-associated mortality [5,6]. The mechanisms of virus and host interaction that dictate the pathogenesis of chronic liver disease, cirrhosis, and HCC are not well understood. This knowledge is essential for developing a biomarker for the early detection of cirrhosis and HCC after an HCV cure.

The replication of the HCV genome occurs predominantly in the endoplasmic reticulum (ER) derived membranes, the most abundant and elaborate membrane-rich organelles [7]. HCV extensively utilizes the ER during all stages of chronic liver disease, leading to cellular stress. Chronic HCV infection leads to the increased accumulation of misfolded proteins in the hepatocytes. The collection of viral proteins and replicative intermediates generates an innate stress response. This maladaptive stress response leaves infected cells vulnerable to additional stress, including metabolic and oxidative stress. The low-level accumulation of misfolded proteins in the ER is cleared by ubiquitin-proteasome degradation, referred to as type I ER-associated protein degradation (type I ERAD). When type I ERAD is not sufficient for reducing chronic stress, the ER initiates a second line of protein degradation process through the induction of autophagy (type II ERAD). The adaptive cellular response through autophagy is activated to reduce stress and improve the survival of the infected cell [8,9]. The cellular stress response is an integral part of liver homeostasis that leads to different types of cell death pathways, which fuel the common tumor suppressor mechanism. Autophagy compromises a set of evolutionally conserved degradation pathways that deliver cytosolic cargoes to the lysosome or endosomes for degradation [10,11]. Autophagy connects two major cell death pathways (necrosis and apoptosis); therefore, it serves a tumor-suppressive role in the liver during chronic HCV infection. Autophagy starts declining during the chronic stage of liver disease, leading to a reduced clearance of misfolded proteins and cellular constituents that results in the development of liver fibrosis and HCC [12]. Nevertheless, how the adaptive cellular response to HCV-microbial stress modulates the autophagy pathway to improve cell survival and HCC development during chronic HCV infection is unknown. This knowledge is essential to prevent virus-associated pathological conditions, such as liver cirrhosis and HCC.

There are three types of autophagy: macroautophagy, chaperone-mediated autophagy (CMA), and microautophagy. These processes can compensate each other to improve cell survival under stress [13,14]. Consistent with these reports, previous publications from this laboratory demonstrated that excessive HCV microbial stress inhibits autophagy by impairing the fusion of the autophagosome with the endosome or lysosome [12,15]. It was observed that CMA is compensatively activated in HCV-infected culture to improve cell survival. However, CMA cannot degrade aggregated misfolded proteins, lipids, and nucleic acids. It is unclear how dysfunctional viral and cellular components are removed from chronically infected cells when autophagic degradation is inhibited.

Extracellular vesicles (EVs) are a heterogeneous group of vesicles released by cells under physiological and pathological conditions. They can be originated through the endosomal pathway from multivesicular bodies (MVBs), or pinched off from the cell membrane. MVBs are membrane-bound organelles generated from the invagination of the late endosomal membrane and intraluminal bodies [16]. They are involved in the transporting, storing, sorting, recycling, and releasing of many substances derived from the Golgi complex, ER, and mitochondria [17,18,19]. They also participate in autophagy through the degradation of organelles, proteins, and RNA [20]. During autophagy, MVBs fuse with an autophagosome, generating a hybrid organelle called an amphisome, which fuses with lysosomes for degradation [21,22,23]. Based on these pieces of evidence, we propose that the extracellular release of misfolded protein aggregates, nucleic acids, molecular chaperones, cytosolic proteins, lipids, and small RNAs through exosomes is an efficient cellular adaptive mechanism to improve cell survival during persistent HCV infection.

This study was performed to test the hypothesis of whether EV release is a part of the adaptive cellular response to microbial stress that promotes virus-cell survival during chronic infection. Our results showed that increased cellular stress during persistent infection impairs autophagic degradation. Impaired autophagic degradation of HCV replicative intermediates promotes the release of EVs and exosomes. The inhibition of EV release by small molecule inhibitors dramatically suppressed virus replication and activated innate antiviral program, leading to interferon (IFN) production. Collectively, our study’s results illuminate an adaptive cellular response to HCV microbial stress that promotes the release of EVs which contribute to the persistent viral infection.

## 2. Materials and Methods

### 2.1. Cell Culture, Antibodies, and Chemicals

Huh-7.5 cells were obtained from Charles M. Rice (Rockefeller University, New York, NY, USA). The Huh-7.5 cell line was maintained in Dulbecco’s Modified Eagle’s Medium (DMEM; Life Technologies, Carlsbad, CA, USA) supplemented with 2 mM L-glutamine, sodium pyruvate, nonessential amino acids, 100 U/mL penicillin, 100 μg/mL streptomycin, and 10% fetal bovine serum (Life Technologies, Carlsbad, CA, USA). Huh-7.5 cells were transfected with a CD63-GFP plasmid (Addgene, Watertown, MA, USA, plasmid # 62964), and multiple stable cell lines expressing (Huh-7.5-CD63-GFP) were prepared using G-418 (Sigma-Aldrich, St. Louis, MO, USA) selection. Huh-7.5 cells were infected with either JFH1-GFP chimera or JFH1-Renilla luciferase chimera HCV using a protocol developed in our laboratory, as previously described [24,25]. A DQ-Red BSA assay (D12051) was acquired from ThermoFisher Scientific. The antibody to NS3 was purchased from Virogen Inc. (Boston, MA, USA). Antibodies to HCV core were purchased from Thermo Scientific (Waltham, MA, USA). Antibodies to β-Actin, LC3B, and CD63 were obtained from Cell Signaling (Beverly, MA, USA). Antibodies to CD9 and p62 were purchased from Santa Cruz Biotechnology (Santa Cruz, CA, USA). Torin1 (475991), Hydroxychloroquine (HCQ) (H0915), MG132 (M7449) and GW4869 (D1692) were obtained from Sigma-Aldrich (St. Louis, MO, USA). Manumycin A (SC200857), Calpeptin (SC202516), Y27632 (SC281642), D-Pantethine (SC252658), and Imipramine (SC207753B) were purchased from Santa Cruz Biotechnology (Santa Cruz, CA, USA).

### 2.2. Western Blotting

Western blotting was performed using a standard protocol established in our laboratory. Infected cells were harvested by the treatment of trypsin- EDTA (Life Technologies, Carlsbad, CA, USA) at different time points and were washed twice with PBS, then lysed in ice-cold RIPA buffer (Sigma-Aldrich, St. Louis, MO, USA) with a protease inhibitor (ThermoFisher Scientific, Waltham, MA, USA) and phosphatase inhibitor cocktail (Sigma-Aldrich, St. Louis, MO, USA). The total protein content of the extract was quantified using NanoDrop^TM^ 2000 (ThermoFisher Scientific, Waltham, MA, USA). Cell lysates (approximately 20 μg of protein) were loaded by SDS-PAGE and transferred into a nitrocellulose membrane (0.45 mm pore size, ThermoFisher Scientific, Waltham, MA, USA). The membrane was blocked using 0.05 g/mL blotting-grade milk powder (Bio-Rad, Hercules, CA, USA) for two hours, then incubated with primary antibody for overnight incubation on an orbital shaker. After overnight incubation, the antigen-antibody complex was visualized with HRP-conjugated goat anti-rabbit or anti-mouse IgG (Cell Signaling, Beverly, MA, USA), then developed with an ECL detection system (Supersignal^TM^ West Pico PLUS, ThermoFisher Scientific, Waltham, MA, USA) using the Bio-Rad ChemiDoc imaging system.

### 2.3. Confocal Microscopy

The relationship of HCV core and CD63-GFP expression was verified by confocal microscopy. Huh-7.5 cells stably transfected with CD63-GFP (Huh-7.5-CD63-GFP) were infected with JFH1-RLuc chimera HCV using a standard protocol [25]. These cells were examined for GFP expression on day 9 and day 21 after virus infection. Cells were incubated for 1 h at 4 °C with HCV core antibody (1:100 dilution) in ice-cold DMEM containing 1% FBS with gentle shaking. Cells were then washed once with DMEM containing 1% FBS and incubated with Texas Red (ThermoFisher Scientific, Waltham, MA) with 1:1000 concentration at 4 °C with shaking for 30 min. Finally, cells were washed twice with DMEM containing 1% FBS and fixed with 4% paraformaldehyde. The cell suspension was then examined for HCV core and CD63-GFP expression using confocal microscopy. In addition, 4′,6-diamidino-2-phenylindole (DAPI) staining was used for nuclear imaging. Finally, the expression of GFP, as well as the HCV core, was monitored using a Nikon A1 confocal microscope. Transfected cells were examined using a fluorescence microscope (Olympus IX73, Tokyo, Japan) at 484 nm for the expression of green fluorescence, 563 nm for the expression of red fluorescence, and 340 nm for DAPI. For each area, two sets of pictures were generated. Then combined pictures was generated by superimposing different fluorescent images with using Olympus cellsSens Dimension version 1.15 software.

### 2.4. Exosome Isolation and Quantification

For EV isolation, infected cells were cultured in exosome depleted medium (ThermoFisher Scientific, Waltham, MA, USA). Culture supernatant was collected at multiple time points and centrifuged at 2000× *g* for 30 min to remove cellular debris. The supernatant was transferred to a new sterile tube and 0.5 volumes of total exosome isolation reagent (for cell culture medium) from Invitrogen were added. Samples were incubated at 4 °C overnight and subsequently centrifuged at 10,000× *g* for 60 min at 4 °C. The supernatant was removed without touching the exosome-containing pellet. The pellet was resuspended in 1× phosphate-buffered saline (PBS) and stored at −20 °C for downstream analysis. The absolute concentration and size distribution of EVs from infected cultures were measured using NanoSight (Model NTA3300 with 532 nm green laser module, Malvern, Worcestershire, UK), which is a laser-based light scattering system [26,27]. The general nanoparticle quantification range was 10^6^ to 10^9^ particles/mL. Exosome pellets were serially diluted 1:100 to 1: 10,000 with particle-free water before starting the analysis. If the particle count was above the detection limit in the initial analysis, we then used the next dilution level. Final exosome concentrations were calculated according to the dilution factor. Exosome count and size were calculated by Nanosight Nanoparticle analysis (NTA) software version 2.3 (Malvern, Worcestershire, UK). For every sample, at least 30 s and three different video image sets were captured.

### 2.5. Cryo-TEM for Exosome Characterization

Transmission Electron Microscopy (TEM) and Cryo-TEM analyses were performed using exosomes purified from cell culture supernatants by ultracentrifugation protocol [28]. Culture supernatants were centrifuged at 1000× *g* at room temperature for 10 min. The supernatants were collected and spun again at 10,000× *g* at room temperature for 30 min to remove cellular debris. The supernatants were filtered through 0.22 μm filters (Sigma-Aldrich, St. Louis, MO, USA), and exosomes were precipitated by ultracentrifugation at 100,000× *g* at room temperature for 2 h (Beckman Ultracentrifuge). The exosome pellet was resuspended in PBS for downstream analysis. Cryo-TEM was performed to demonstrate the purity and size of exosomes released from the HCV-infected cell culture using an FEI G2 F30 Tecnai TEM operating at 150 kV. The exosome samples were prepared on a lacey carbon-coated copper grid (200-mesh, electron microscopy sciences) using an automated plunging station (FEI Vitrobot). The sample solution was applied to the grid. The excess liquid was blotted by attached blotting papers for 2 s to produce a thin sample film that was immediately vitrified by plunging to liquid ethane. The grid with the sample cryogenically immobilized was transferred onto a single tilt cryo-specimen holder for imaging.

### 2.6. Transmission Electron Microscopy (TEM)

Uninfected and infected Huh-7.5 cells on days 9 and 21 were harvested using trypsin-EDTA. Cell pellets were washed with PBS and then suspended in 3% glutaraldehyde fixative (Sigma-Aldrich, St. Louis, MO, USA). Cell pellets were fixed in 1% osmium tetroxide and dehydrated with an ethyl alcohol series. Samples were infiltrated and embedded in eponate-12 resin and polymerized at 60 °C for 24 h. Thin sections (70 nm) of the samples were placed on copper grids. Cells were examined using a G2 F30 Tecnai TEM at 200 kV. We captured cytoplasmic areas of 10 different cells under the grid, and the number of autophagic vacuoles (AVs) and multivesicular bodies (MVBs) was counted in uninfected, early, and late-infected Huh-7.5 cells.

### 2.7. MTT Proliferation/Viability Assay

Cells were counted with an EVE automated cell counter (NanoEnTek, Seoul, Korea). Huh 7.5 cells were seeded in 96 well plates with 7500 cells/well density. Each treatment group was prepared in triplicate. After 72 h, 20 µL MTT solution (5 mg/mL) was added per well into the culture medium. The plate was incubated for 3.5 h at 37 °C in a culture hood. After removing the medium, 150 µL MTT solvent was added per well, and the plate was covered with tinfoil and incubated on an orbital shaker for 15 min. Absorbance measurements were performed at 595 nm with a reference filter of 655 nm in iMark Microplate Reader (BIORAD Hercules, CA, USA). MTT solution and MTT solvent were prepared according to a previously described protocol [29].

### 2.8. Enzyme Linked Immunosorbent Assay (ELISA)

After exosome inhibitor treatment, IFNL1 protein concentration in cell culture supernatants was measured using Human IL-29 ELISA kit (Invitrogen, ThermoFisher, Waltham, MA, USAcat#88–7296-22). In brief, wells were incubated with 50 µL of culture supernatant, and the remaining steps were carried out using the Sandwich ELISA protocol. The plate was read at 450 nm. The concentration of IFNL1 was calculated using the standard curve of the internal control supplied in the ELISA kit.

### 2.9. Statistical Analysis

Statistical analysis was performed using by GraphPad Prism software version 8 (GraphPad Software, Inc., La Jolla, CA, USA). All experiments were performed 3 independent times with fresh cultures of cells each time to obtain 3 replicates. Because Huh-7.5 is the only cell line in which these experiments can be done, this approach provides measurements with the maximum possible biological independence. However, in order to prevent a violation of the independency assumptions, 2-tailed paired *t* tests were used to compare means of variables. The statistical significance was shown as * *p* < 0.05, ** *p* < 0.01, *** *p* < 0.001.

## 3. Results

### 3.1. Autophagic Degradation and Exosome Release Supports Virus-Cell Survival during Persistent HCV Infection

**Figure 1:**
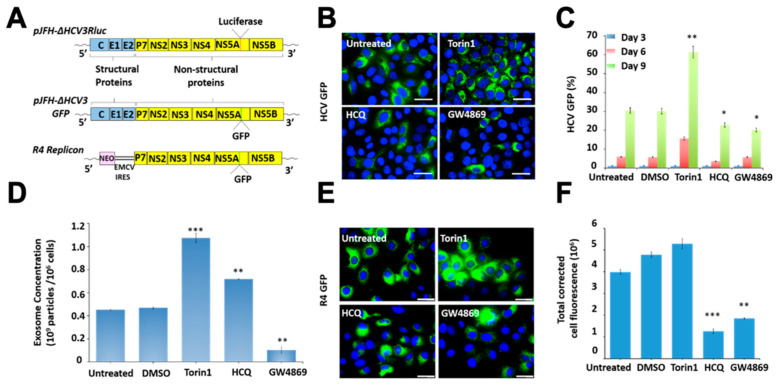
Illustrated is the impact of autophagy induction, lysosomal degradation, and exosome release on virus-cell survival in persistently infected HCV culture. (**A**) Schematic of the construct used for HCV infection and the sub-genomic replicon cell line. The impact of autophagy induction, lysosome inhibitor (HCQ), and exosome inhibitor (GW4869) on HCV GFP expression by flow analysis. Huh-7.5 cells were infected at the HCV GFP virus at MOI 0.01. Infected cells on days 3, 6 and 9 were treated with 2 rounds of Torin1 (100 nM), HCQ (10 μM), or GW4869 (10 μM). (**B**) Fluorescence image of HCV-GFP expression. (**C**) Quantification of HCV-GFP by flow analysis at 3, 6 and 9 days after treatments. (**D**) Nanoparticle tracking analysis (NTA) shows the impact of Torin1, HCQ and GW4869 treatment on extracellular vesicle release in R4GFP replicon cells. (**E**) Fluorescent microscopy images of untreated R4GFP replicon cells and treated with Torin1 (100 nm), HCQ (10 μM) and GW4869 (10 μM) for 72 h. (**F**). Fluorescence intensity measurements representing the GFP expression of replicon cell culture. * *p* < 0.05, ** *p* < 0.01, *** *p* < 0.001. Error bars represent the standard deviation (SD) of 3 measurements.

Initially, we tested the impact of autophagy, lysosomal degradation and exosome release on HCV replication and dissemination. We used an infectious full-length GFP reporter-based chimera HCV virus and sub-genomic replicon cell culture models, established in our laboratory (Figure 1A). Huh-7.5 cells were infected with HCV-GFP-chimera virus and treated with the autophagy inducer (Torin1), lysosomal inhibitor (HCQ), or the MVB inhibitor (GW4869) for 72 h. The MTT assay determined the optimal dosage for each drug: 100 nM for Torin1, 10 μM for HCQ, 10 μM for GW4869 (Figure S1). The amount of GFP-positivity was examined under fluorescence microscopy and then quantified by flow analysis at different time points. As expected, we found that the induction of autophagy by Torin1 treatment increased HCV replication and spread (Figure 1B,C). Inhibition of lysosomal degradation by HCQ treatment decreased the percentage of HCV-positive cells. Inhibition of exosome release by GW4869 treatment inhibited HCV replication (Figure 1B,C). The impact of autophagy induction, HCQ, and GW4869 treatment on extracellular vesicle release was quantified by NTA. It was found that Torin1 and HCQ treatment increased extracellular vesicles, whereas GW4869 inhibited extracellular vesicle release (Figure 1D). We examined the impact of Torin1, HCQ, and GW4869 treatment on intracellular HCV RNA replication in a sub-genomic HCV replicon (R4GFP) cell line that did not produce the virus since it lacked the structural genes. This cell line is resistant to interferon alpha (IFNA) but sensitive to interferon lambda 1 (IFNL1). IFN-resistant R4GFP cells were treated with either Torin1, HCQ or GW4869 for 72 h. The expression of GFP was quantified by flow analysis and fluorescence microscopy (Figure 1E,F). The results show that, while autophagy induction supports replication of HCV sub-genomic RNA, HCQ and GW4869 treatment inhibit replication. Inhibition of lysosomal degradation and exosome release decreased HCV replication. We found HCQ that inhibits lysosomal degradation shows increased exosome release. Taken together, our data using both models suggest that autophagy induction promotes HCV infection and spread, which is consistent with an earlier study on poliovirus, where autophagy induction correlates with viral replication [30].

### 3.2. Persistent HCV Infection Inhibits CD63-Mediated Autophagic Endosome-Lysosomal Degradation

**Figure 2:**
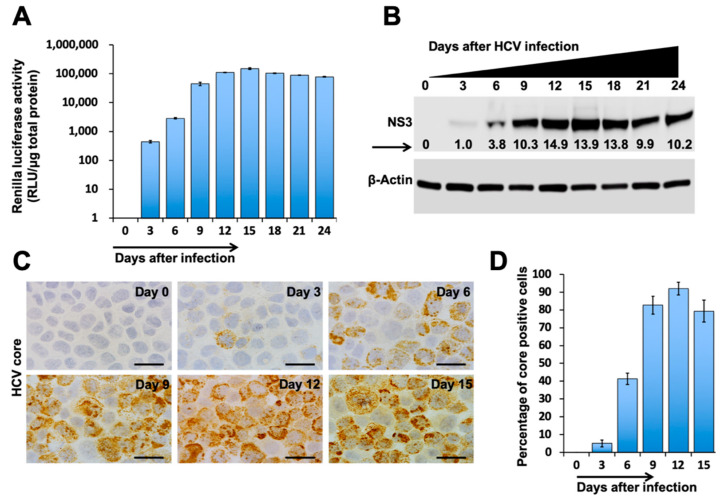
HCV replication in the Huh-7.5-CD63-GFP cell line. Huh 7.5 cells were infected with HCV-Renilla luciferase virus (JFH1-V3-Rluc) at an MOI of 0.1 by overnight incubation. The next day, cells were incubated with fresh media with 10% FBS. The infected cells were maintained with regular medium change at a three-day interval. (**A**) Renilla luciferase activity of infected cells. (**B**) Western blot analysis shows a time-dependent increase in the expression of NS3 protein in infected culture. (**C**) The expression of HCV core protein by immunostaining over 12 days (Original magnification, ×60). (**D**) Percentage of HCV core positive cells at 0, 3, 6, 9, and 12 days. Error bars represent the standard deviation (SD) of three experiments.

Tetraspanins (TSPAN) CD9, CD63, and CD81 are enriched in the membrane of exosomes, which are often used as exosomal markers [31]. The TSPAN protein, CD63, plays a crucial role in endosomal cargo sorting and EV production [32,33]. To understand the impact of autophagy–lysosome pathways regulating MVBs degradation and exosome release, we developed a persistent HCV replication model using Huh-7.5-CD63-GFP liver cell culture. The replication of HCV-genotype 2a virus (JFH-deltaV3-Rluc chimera) in CD63-GFP cells was studied by the measurement of luciferase activity for over one month. Consistent with our previous report, we were able to demonstrate the high-level replication of this chimera virus in Huh-7.5 cells and Huh-7.5 with CD63-GFP (Figure 2A). We found that most of the cells in culture at day 9 show high-level expression of NS3 by Western blotting (Figure 2B) and viral core protein expression by immunostaining (Figure 2C). The number of core protein expressing cells quantified by ImageJ software can be seen in Figure 2D.

**Figure 3:**
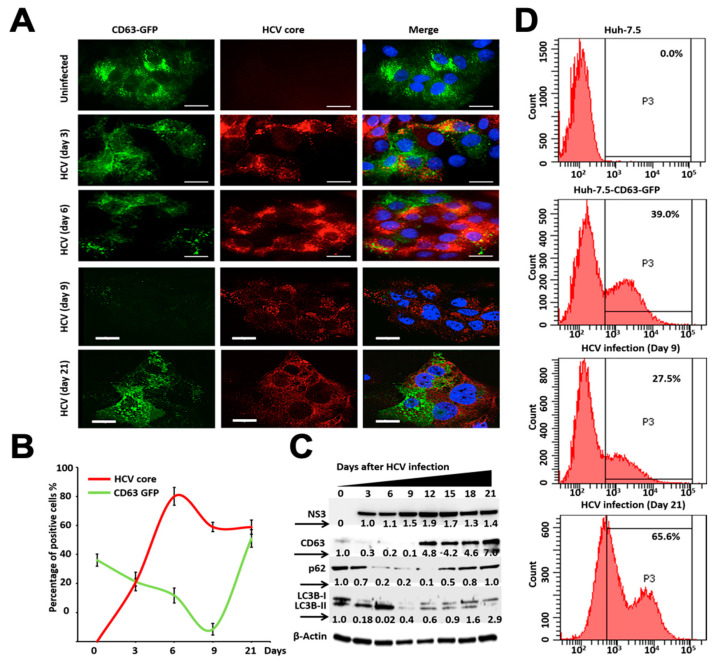
Impact of persistent HCV replication on the expression levels of CD63-GFP protein. (**A**) Representative image from confocal microscopy showing expression of CD63-GFP and HCV core in the infected cells at days 3, 6, 9 and 21. Left panel illustrates green fluorescence from CD63-GFP stable cells. Middle panel shows red fluorescence from HCV core expression. Right panel shows colocalization of green and red fluorescence images. The GFP fluorescence expression decreased from day 3 to 9 in infected culture, whereas they are detectable in day 21 in the infected culture. (**B**) Quantification of green fluorescence and red fluorescence positive cells by ImageJ software. (**C**) Western blot analysis showing the expression of HCV NS3, CD63-GFP, P62 and LC3B-I/II levels after HCV infection over 21 days. (**D**) Quantification by flow analysis shows early-stage HCV infection (day 9) promotes degradation, (39% to 27.5%), whereas the late-stage of infection accumulates CD63-GFP (39% to 65.6%). Error bars represent the standard deviation (SD) of three experiments.

We examined the impact of autophagy inhibition on MVB formation and degradation by quantifying CD63-GFP expression. Huh-7.5-CD63-GFP cells were infected with HCV, and the expression of GFP and HCV core expression was measured at 3, 6, 9, and 21 days by confocal microscopy (Figure 3A,B). The impact of HCV replication in the Huh-7.5-CD63-GFP cells on cellular autophagy flux and MVB degradation was confirmed by the measurement of NS3 and CD63 expression by Western blot analysis (Figure 3C). Western blot analysis showed that autophagic flux (p62, LC3BI/II ratio) was gradually decreased due to HCV replication until day 9. The levels of p62 and the LC3BI/II ratio were increased after day 12, suggesting that the late stage of persistent HCV infection inhibits autophagic flux (Figure 3C). These results are consistent with data shown in our previous publication showing that persistent HCV replication inhibits autophagy [15]. The HCV-induced effect on CD63-GFP expression was verified by flow analysis on day 9 and day 21. We found that the early stage of persistent HCV infection efficiently degraded CD63-GFP expression (39% decreased to 27.5%), whereas late-infected culture showed increased expression (39% to 65.6%) (Figure 3D). All these results indicate that the early stage of HCV replication degrades MVBs, and that the late-stage of persistent HCV replication inhibits the degradation of CD63-GFP.

### 3.3. Autophagy Induction Promotes Degradation of MVBs

**Figure 4:**
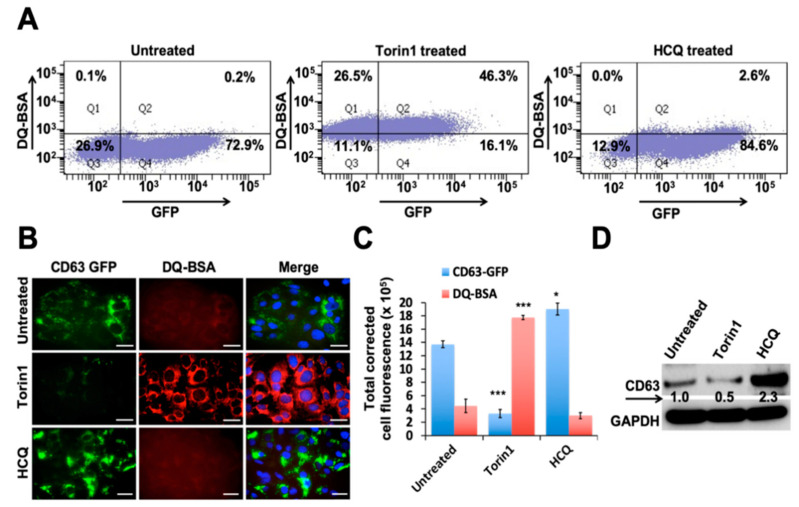
The impact of autophagic degradation of CD63-GFP chimera in uninfected Huh-7.5 cells. Huh-7.5 cells were treated with DQ-BSA and then treated with either autophagy inducer (Torin1- 200 nM) or lysosomal inhibitor (HCQ- 10μg) for 24 h. (**A**) Autophagy induction degrades CD63-GFP fluorescence, whereas lysosomal inhibitor accumulates CD63-GFP. (**B**) Demonstrated are the fluorescent images of Huh-7.5 cells transfected with CD63-GFP and treated with either Torin1 or HCQ. (**C**) Shown is the quantification of mean fluorescent intensity (MFI) data by three separate analyses. * *p* < 0.05, *** *p* < 0.001. Error bars represent the standard deviation (SD) of three experiments. (**D**) Western blot verifies autophagic regulation of CD63 expression.

MVBs are membrane-bound organelles that belong to endosomal pathways. They play a major role in cellular metabolism such as transporting, storing, sorting, recycling, and releasing protein, lipids, and small RNAs derived from the Golgi, ER, and mitochondria. We examined whether autophagy induction or inhibition through small molecule drugs alters the expression of CD63-GFP without any viral infection. Huh-7.5-CD63-GFP cells were treated with an autophagy inducer (Torin1) and lysosome inhibitor (HCQ) for 24 h. Torin1 is a potent and selective ATP-competitive inhibitor of mTOR (mechanistic target of rapamycin) kinase. HCQ is an alkalinizing drug that inhibits lysosomal degradation by increasing pH. The next day, cells were incubated with bovine serum albumin derivatives conjugated to a self-quenched fluorophore (DQ-BSA), which is bovine serum albumin labelled with a red fluorophore (RFP) for one hour, and then the amount of GFP and RFP expression was quantified by flow analysis. Autophagy induction in Huh-7.5-CD63-GFP cells by Torin1 treatment increased CD63-GFP degradation, since the percentage of GFP positive cells decreased (72.9% to 16.1%), whereas the rates of DQ-BSA positive red fluorescence cells were increased (0.2% to 46.3%). Autophagy inhibition by HCQ treatment impaired lysosomal degradation, leading to an increase in CD63-GFP positive cells (72.9% to 84.6%) and only 2.6% red fluorescence positive cells by flow analysis (Figure 4A). A morphological evaluation of cells treated with Torin1 and HCQ was performed under fluorescence microscopy and showed that autophagy induction degraded, but autophagy inhibition accumulated, CD63-GFP (Figure 4B). Quantification of these data from three separate investigations revealed that the autophagy inducer Torin1 degrades CD63-GFP, whereas HCQ treatment accumulates its expression (Figure 4C). DQ-BSA fluorescence was strong in Torin1-treated culture, but not in the HCQ-treated culture. The impact of autophagy modulation by the treatment of Torin1 on the degradation of MVBs protein CD63-GFP was verified by a Western blot analysis (Figure 4D). These results are consistent with our observation in HCV-infected culture showing that autophagy inhibition promotes accumulation of CD63-GFP due to their impaired lysosomal degradation at the level of autophagosome-lysosome fusion.

### 3.4. Persistent HCV Replication Decreased Autophagic Vacuoles and Autophagosome-Lysosome Fusion without Affecting Lysosomal Activity

**Figure 5:**
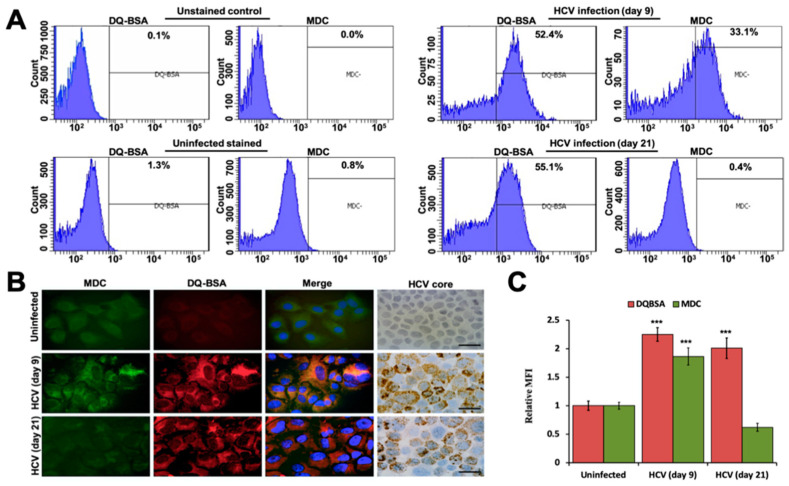
Huh-7.5 cells were infected with the Renilla luciferase virus. The impact of HCV replication on autophagy vacuoles (AVs) formation was assessed at early and late-stage of infection. (**A**) Flow-based quantification of AVs in early and late-infected culture after MDC staining or DQ-BSA. Days 9 and 21 of infected culture were incubated with 0.05 mmol/L MDC or DQ-BSA for 10 min. After this treatment, cells were washed with phosphate-buffered saline (PBS), suspended in 0.5 mL of PBS, and then subjected to flow analysis. Left panel shows unstained controls. Right panel shows flow analysis of MDC or DQ-BSA treatment of 9- and 21-day infected cells. Only day-21 infected culture shows reduced MDC fluorescence. DQ-BSA degradation by lysosome culture shows comparable fluorescence between early and late-infected culture. (**B**) Illustrated is the fluorescence microscopy images of early and late-infected culture after staining with both MDC and DQ-BSA. (**C**) Graph presenting mean fluorescence intensity (MFI) of DQ-BSA and MDC. *** *p* < 0.001 versus uninfected. Error bars represent the standard deviation (SD). *** *p* < 0.001 versus uninfected.

The impact of HCV replication on autophagosome-lysosome (autolysosome formation) was verified using alternative approaches. Monodansylcadaverine (MDC) was used previously for the labeling of active AVs. DQ-BSA was used to quantify lysosomal protease activity. These two reagents were used to measure autophagy between early (day 9) and late-infected culture (day 21) by flow cytometry. We found that the number of DQ-BSA positive cells was comparable between early and late-infected cultures (52.4% vs. 55.1%), whereas there was a marked difference in the MDC positive fluorescence observed between early and late-infected culture (33.1% vs. 0.4%) (Figure 5A). Uninfected Huh-7.5 cells and HCV-infected Huh-7.5 cells without any DQ-BSA or MDC treatment did not show any positive staining. These results were confirmed by the visualization of infected cells under fluorescence microscopy. The only early stage of persistent HCV-infected cells led to AVs that stained with MDC. Similar staining was not observed in late-infected culture, suggesting the absence of AVs in late-infected culture (Figure 5B). The red fluorescence staining displayed a comparable active lysosomal protease activity between the early stage and late stage of the persistently infected culture. A statistical analysis of the effects of three separate experiments proved that persistent HCV replication results in significant inhibition of autophagy induction without compromising the cellular lysosomal degradation (Figure 5C).

### 3.5. Ultrastructural Analysis of Huh-7.5 Cells Infected with HCV

**Figure 6:**
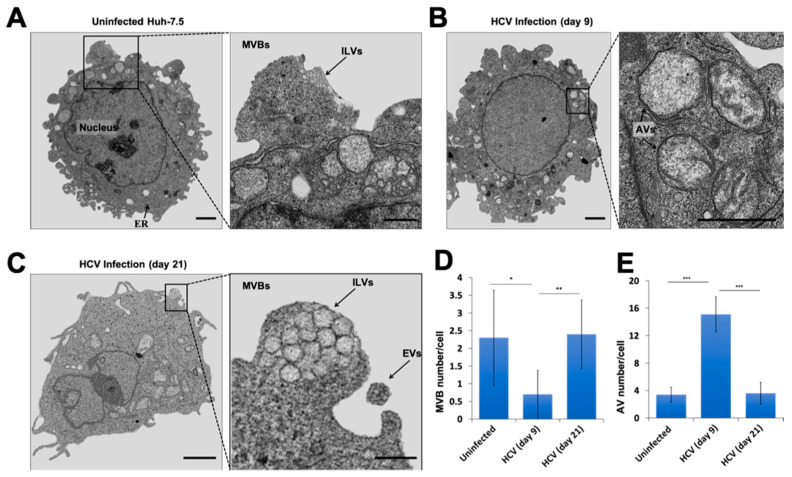
Electron microscopy images of extracellular vesicles (EVs), intraluminal vesicles (ILVs), and autophagic vacuoles (AVs) on different days of infection. Illustrated are the electron microscopy images of MVBs accumulation, which was more frequent in late-infected HCV culture than early infected culture. (**A**) MVBs were more frequently found in uninfected Huh-7.5 tumor cell lines. (**B**) MVBs are less frequent in day 9 infected culture due to increased autophagy. AVs with partial degradation of double membranes are seen in day 9 infected culture. (**C**) Persistent HCV infection accumulates MVBs. AVs are not detected in day 21 infected culture. (**D**) Cytoplasmic areas of 10 different cells were captured under the grid. The number of MVBs were counted in uninfected, early and late-infected Huh-7.5 cells. (**E**) The number of AVs was counted in uninfected, 9- and 21-day infected culture. * *p* < 0.05, ** *p* < 0.01, *** *p* < 0.001. Error bars represent the standard deviation (SD) of measurements.

The presence of HCV-induced AVs and MVBs were compared in the early and late stages of HCV infection by transmission electron microscopy (TEM). Cytoplasmic areas of 10 different cells were imaged under the grid; the numbers of autophagosomes and MVBs were counted. The number of AVs per field per cell was compared among uninfected, early stage of infection (day 9), and late-stage of infection (day 21) (Figure 6A–C). The number of MVBs were normalized back to control levels, after a reduction at day 9 (Figure 6D). In contrast, large numbers of AVs with the partial dissolution of the double membrane due to lysosome fusion were present in the early infected culture (Figure 6E). Ultrastructural analysis was consistent with liver cell imaging studies of persistently infected HCV culture.

### 3.6. Persistent HCV Infection Promotes Release of Extracellular Vesicles and Exosomes

**Figure 7:**
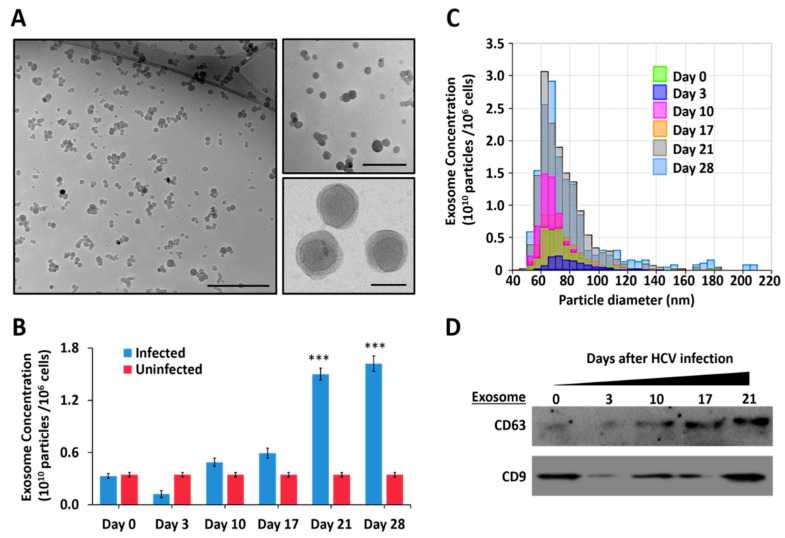
Persistent HCV infection promotes exosome release. (**A**) Transmission electron microscopy (TEM) of negatively stained morphology of exosomes purified from late-infected culture supernatants. (**B**) NTA analysis shows exosome release increased in persistently infected HCV culture. *** *p* < 0.001. (**C**) A very uniform size of exosomes is released in HCV-infected culture. Error bars represent the standard deviation (SD) of three different measurements. (**D**) An equal amount of exosome lysates was loaded on to SDS-PAGE. The presence of various endo-lysosomal cargoes was detected by Western blot analysis. CD9 and CD63 expressions confirmed the purity of exosome preparation.

We studied the effect of autophagy on the release of EVs and exosomes in a persistently infected HCV cell culture model. For this purpose, we used Huh-7.5 cells infected with HCV-luciferase chimera virus. Infected cells were cultured in the exosome-depleted cell culture media. Cell-free supernatant was collected at different time intervals, and exosomes were isolated from 1 mL of cell culture media by total exosome isolation kit (Invitrogen) and were characterized through multiple approaches. Cryo-TEM examination confirmed the purity and size of exosomes released from the HCV-infected cells (Figure 7A). The NTA was used to count the absolute particles secreted into the culture supernatants over 28 days post-HCV infection. We found that exosome release gradually increased with time in persistently infected HCV culture (Figure 7B). We found that infected cultures release very uniform size exosomes 60–80 nm (Figure 7C). A Western blot analysis found enrichment of TSPANs (CD63 and CD9) in the exosomes isolated from HCV-infected culture over time (Figure 7D).

### 3.7. Release of Extracellular Vesicles Promotes Virus Replication during Persistent HCV Infection

**Figure 8:**
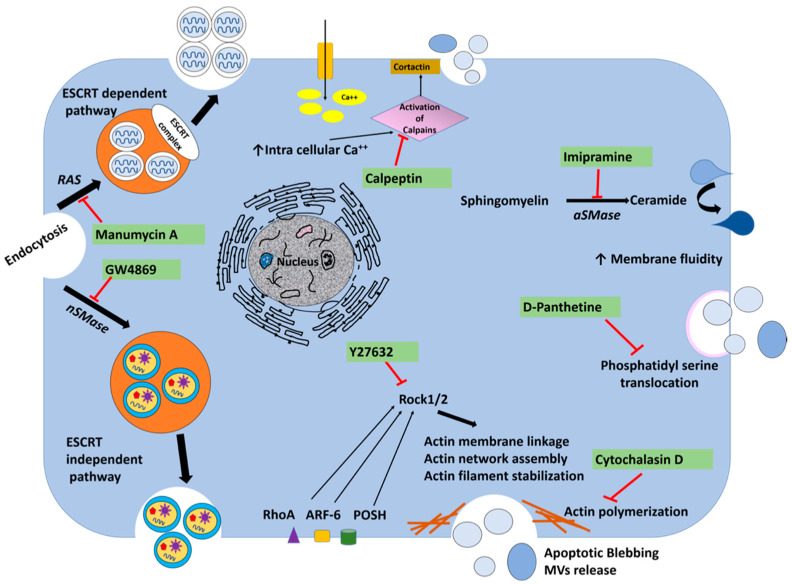
The action mechanism of drugs used to inhibit extracellular vesicle release. Extracellular vesicles originate from the endosomal pathway or pinch off from the cell membrane. Exosomes are produced from MVBs by ESCRT-dependent and ESCRT-independent pathways. While manumycin A inhibits exosome dependent pathway, GW4869 inhibits exosome independent pathway. Microvesicle biogenesis is modulated by lipids and cytoskeletal proteins. Lipid rafts and cholesterol play an important role in the budding of cell membranes. Enzymes involved in the transfer of lipid from one leaflet of the cell membrane to the other are potentially targeted to inhibit exosome release. Calpeptin is a family of calcium-dependent neutral cytosolic cysteine protease inhibitors used as a micro-vesicular inhibitor. Y27632 is a competitive inhibitor of both ROCK1 and ROCK2 and is able to compete with ATP in binding to the catalytic site of these kinases. This compound inhibits microvesicle release by blocking these two proteins. This compound reorganizes the cytoskeleton and mediates cellular contractility by regulating the activity of actin filaments. Imipramine is a well-known antidepressant that promotes membrane fluidity by inhibiting acid sphingomyelinase (aSMase), therefore preventing the generation of microvesicles. D-Pantethine inhibits cholesterol synthesis as well as fatty acid synthesis as the fluidity of the cell membrane is important during membrane bi-layer reorganization and microvesicle formation. This drug blocks the translocation of phosphatidylserine to the outer surface membrane, which is an essential step of microvesicle formation. Cytochalasin D is an alkaloid produced as a toxin by many fungi. This compound binds the edges of actin filaments to prevent actin polymerization. Actin polymerization is essential for the formation of membrane-derived microvesicles and their intracellular movement.

Virus infection also releases vesicles that originate either from the intraluminal vesicles (ILVs) of multivesicular endosome, called exosomes, or microvesicles which are directly budding from the plasma membrane. Microvesicle biogenesis is modulated by membrane lipids and the organization of the peripheral actin cytoskeleton, both known to alter membrane fluidity, membrane invasion, and fusion [34]. Actin polymerization and myosin contraction are involved in the biogenesis of microvesicle formation and their intracellular movement and cargo transport [35,36]. MVBs primarily fuse with lysosomes for degradation and degradation of viral double-stranded RNA replicative intermediates. The late stage of persistent HCV infection prevents MVBs degradation, therefore allowing exosome release. We investigated the impact of inhibition of EVs and exosome release on HCV replication in the infected cells and replicon model. A number of pharmaceutical agents were selected to inhibit EVs released, and their mechanisms of action are shown in Figure 8.

**Figure 9:**
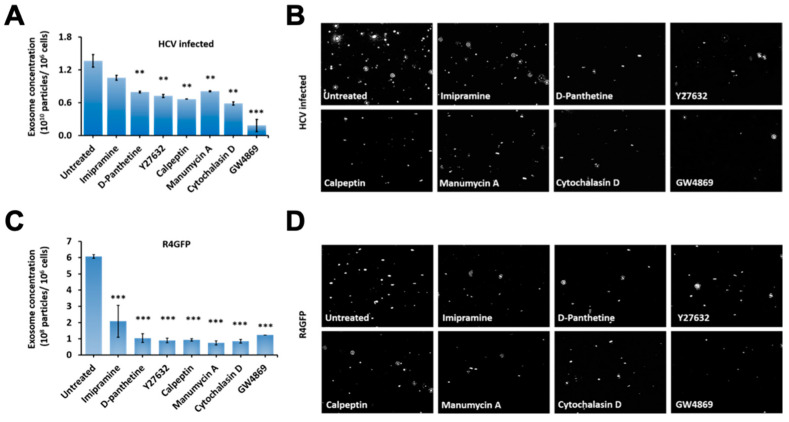
The extracellular vesicle (EV) release from infected cells and sub-genomic R4GFP replicon cells treated with different drugs individually. (**A**) Late-infected (day 21) HCV culture was treated with each inhibitor at their viable concentration for 72 h (Imipramine 10 µM, D-Pantethine 100 µM, Y27632 10 µM, calpeptin 30 µM, manumycin A 2 µM, and cytochalasin D 2 µM). We found all drugs show significantly inhibited release of EVs. The graph demonstrates the difference in exosome concentration between treated and untreated groups. ** *p* < 0.01, *** *p* < 0.001. (**B**) Visual assessment of microvesicles during movement in liquid phase in the untreated control cells and treatment with different inhibitor. (**C**) Bar chart shows the mean EV concentrations of R4GFP cells which were treated with individual compound for 72 h. *** *p* < 0.001. (**D**) Images of particles Brownian motion in liquid suspension.

Seven different compounds with known mechanisms of action were selected. An MTT assay determined their cellular toxicities using Huh7.5 cells (Figure S2). Persistently HCV-infected Huh-7.5 cells were treated with each drug (Imipramine 10 µM, D-Pantethine 100 µM, Y27632 10 µM, calpeptin 30 µM, manumycin A 2 µM, cytochalasin D 2 µM and GW4869 at 20 μM) for 72 h, and then extracellular vesicle released to the cell culture supernatants were quantified by NTA. We found that almost all of the inhibitors used in our assay decreased extracellular vesicle release (Figure 9A). The NTA was used to capture the movement of particles in the liquid stage. Brownian motion of the purified exosomes from the infected culture with and without treatment of different inhibitors was recorded (Figure 9B). Exosome release was also decreased when the R4GFP sub-genomic replication cell line was treated with each inhibitor (Figure 9C,D). The inhibition of extracellular release was more prominent in the sub-genomic replicon cell line as compared to the infected cell.

**Figure 10:**
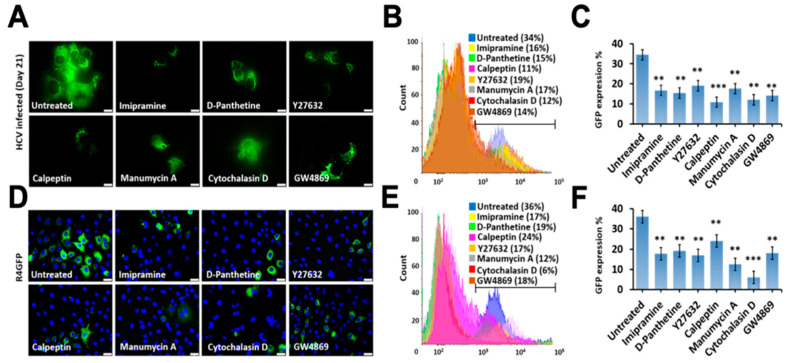
Impact of extracellular vesicle (EV)/exosome inhibition on HCV replication in HCV-GFP-infected cells and R4GFP sub-genomic replicon model. Infected cells or replicon cells were treated with individual drugs at their viable concentration for 72 h (Imipramine 10 µM, D-Pantethine 100 µM, Y27632 10 µM, calpeptin 30 µM, manumycin A 2 µM, and cytochalasin D 2 µM). Cells were examined under fluorescence microscopy and then GFP expression was quantified by flow cytometry. (**A**) The impact of EV release on HCV-GFP expression in the late-stage infected culture. (**B**) Flow analysis of HCV-GFP cells treated with a different inhibitor. We analyzed 10,000 cells per measurement. (**C**) The graph shows the comparison of GFP expression in late-stage HCV infected culture (day 21) treated with different EV inhibitors. ** *p* < 0.01, *** *p* < 0.001. (**D**) R4GFP cells were treated with individual compound for 72 h and GFP expression was assessed by fluorescence microscopy. (**E**) Flow analysis of R4GFP cells treated with different inhibitors. (**F**) The bar chart represents the percentages of GFP expression in R4GFP cell culture treated with EV inhibitors. ** *p* < 0.01, *** *p* < 0.001.

In the next step, we determined whether inhibition of EV release by specific inhibitor treatment at viable concentrations could affect replication. The impact of inhibiting exosome release on HCV replication was examined using late-stage infected culture on day 21. Initially, the effect of inhibiting EV release on host and virus survival was examined using an infectious full-length GFP reporter-based chimera HCV virus. Huh-7.5 cells were infected with HCV-GFP chimera virus and, on day 21, infected cells were then treated with individual inhibitors for 72 h. The number of GFP-positive cells was examined under fluorescence microscopy (Figure 10A) and then quantified by flow analysis (Figure 10B,C). These data show that inhibition of EVs decreased HCV replication by more than 50%. Among all inhibitors, the inhibition of actin polymerization and GW4869 had the strongest inhibitory effect on HCV replication in the infected culture. In the next step, we performed a similar analysis to determine the impact of inhibiting extracellular release on intracellular HCV RNA replication using a stable R4GFP replicon cell line. For this purpose, R4GFP cells were treated with each drug for 72 h. The antiviral effect was determined by examining GFP expression (Figure 10D), and then by flow analysis (Figure 10E,F). All these data from persistently infected and R4GFP replicon models suggest that blocking EVs’ release indeed decreases HCV replication.

**Figure 11:**
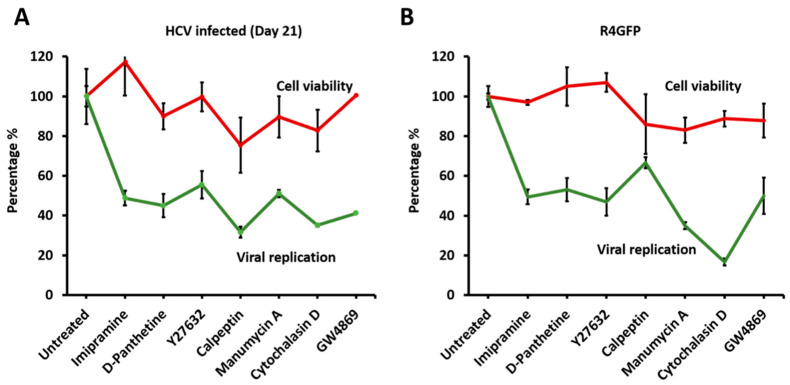
The impact of extracellular vesicle (EV) blockage on virus and cell survival. The percentage of HCV positive cells and cell survival of each compound used in our experiments was compared to assess the benefit of virus-f due to EV blockage. (**A**) Represents data obtained from Huh-7.5 cells infected with HCV-GFP chimera virus. (**B**) Represents R4GFP sub-genomic replicon cell line.

The effect of the EV inhibitor treatment on the viability of late-infected HCV culture and stable sub-genomic replicon cell lines was studied after 72 h. The cell viability results, and antiviral efficacy results were compared to determine whether blocking the release of EVs is conducive for virus replication or survival of infected cells. The cell survival and antiviral efficacy of each drug was compared in infected and replicon cell culture models (Figure 11). It appears that inhibition of EVs release has dramatic effect on HCV RNA replication. Cell viability was not affected in the concentration of drugs used in this assay.

We then examined the impact of exosome inhibitors on HCV replication at an early stage of HCV infection. Huh-7.5 cells infected with the JFH-GFP virus on day 3 were treated with a similar concentration of each inhibitor for 72 h. The HCV-GFP expression was measured by fluorescence microscopy and quantified by flow cytometry (Figure S3). These analyses show that only cytochalasin D, imipramine, GW4869 and D-Pantethine show some antiviral effects in the early infected culture. Among those, cytochalasin D showed the strongest inhibitory effect on viral replication. This is probably because cytochalasin D affects all kinds of cellular processes involved in exosome biogenesis, starting with membrane curvature, vesicle formation, and vesicle movement, because all these processes require actin polymerization. Additionally, imipramine and D-Pantethine likely affect the whole secretory pathway (which is strongly dependent on phosphatidyl serine, sphingomyelin, ceramide, and other lipids), and the HCV replication organelles are derived from, or are part of, the secretory pathway. GW4869 that inhibits the ESCRT independent pathway appears to be important in HCV replication.

### 3.8. Inhibition of Extracellular Vesicles Induced Innate Antiviral Response in HCV Culture through Interefron-Lambda (IFNL1) Production

**Figure 12:**
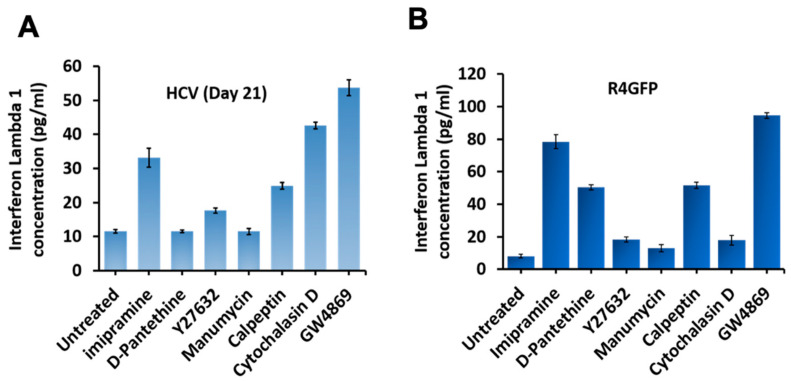
Impact of extracellular vesicle (EV) inhibition of the innate antiviral program through interefron lambda 1 (IFNL1). Late-infected HCV culture were treated with a non-toxic dose of EV inhibitors (Imipramine 10 µM, D-Pantethine 100 µM, Y27632 10 µM, calpeptin 30 µM, manumycin A 2 µM, and cytochalasin D 2 µM). After 72 h, production of IFNL1 was examined in the supernatants by ELISA. (**A**). The cell culture supernatants were examined for the secretion of IFNL1 in peristsently infected HCV culture. (**B**). The levels of IFNL1 in the supernatants of R4GFP cells.

HCV replication accumulates replicative intermediates; viral proteins, therefore, activate intracellular pattern recognition receptors (PRRs). A previous study by Grünvogel et al. [37] showed that double-stranded HCV replicative intermediates (negative-strand RNA) are released through EVs or exosomes. They showed the inhibition of EVs or exosomes leads to the accumulation of the HCV replicative intermediate and, therefore, to the activation of the innate antiviral program. We examined whether the activation of the innate antiviral program through IFN production is the reason for antiviral suppression. A previous publication from this laboratory showed interferon lambda induces potent antiviral response against HCV. In this study, we showed HCV induced ER stress and autophagy response that degrades the interferon alpha and beta receptor subunit 1 (IFNAR1), whereas the expression of interferon lambda receptor 1 (IFNLR1) was not altered [25]. This provides an explanation why interferon-alpha is not effective in clearing HCV infection. We showed that IFNL1 inhibits HCV replication in IFNA-resistant cells, suggesting that the IFNL axis could play an essential role in inducing HCV clearance [38]. R4GFP cells are resistant to IFNA since they express truncated IFNAR1. For this reason, levels of IFNL1 production were measured in the cell supernatants of both infected and R4GFP cells by ELISA. We found IFNL1 expression was increased in R4GFP, as well as HCV infected culture when EV secretion was inhibited (Figure 12A,B). These results provide an explanation as to why inhibiting release of EVs decreased HCV replication.

Taken together, all these data suggest that inhibition of EV release suppresses HCV replication in infected cells, as well as in replicon cell line. These results suggest that EV release is critical to sustain persistent HCV replication. Our results indicate that inhibiting EVs has minimal impact on cell viability but decreased the replication of HCV significantly in both the models. Blocking EVs’ release activates the innate antiviral program through the induction of interefron lambda production. Our data support the conclusion that exosome release supports virus replication during persistent infection by escaping the innate antiviral response.

## 4. Discussion

Autophagy occurs at basal levels in every cell, including hepatocytes, under non-pathological conditions. Hepatic autophagy levels increased several folds after HCV infection to alleviate microbial stress associated with virus replication. Autophagy levels also increased to meet the metabolic demands associated with virus infection to generate ATP, amino acids, sugar, and fatty acids. Data presented in this study are consistent with previous reports of other investigators, suggesting that autophagy induction is beneficial for HCV replication [30,39]. Initially, we examined the role of autophagy induction, extracellular vesicle release, and lysosomal degradation in HCV replication. We found that autophagy inducer Torin1 promoted HCV replication and extracellular vesicle release, as well as lysosomal DQ-BSA degradation. The importance of lysosomal degradation and extracellular vesicle release in HCV replication is supported by the results of HCQ and GW4869 treatment. For example, the compound HCQ inhibits lysosomal degradation and not the extracellular vesicle release inhibited by HCV replication, suggesting that lysosomal degradation is important for sustaining HCV replication. Likewise, GW4869 inhibits extracellular vesicle release, which inhibits replication. All these results indicate that autophagy-induced lysosomal degradation and extracellular vesicles release are two important cellular events required for sustaining HCV replication.

Some researchers, including our laboratory, showed that HCV infection could inhibit autophagy at the level of impaired autophagic degradation. Sir et al. [40] demonstrated that adaptive cellular response to HCV infection induces UPR accumulated through incomplete autophagosomes. Autophagy is impaired due to inefficient fusion between autophagosomes and lysosomes. The autophagy process is also impaired in cells replicating sub-genomic HCV replicon. The accumulation of large aggregates with aberrant cytoplasmic vacuole formation impairs autolysosome maturation [41]. Our laboratory showed that persistent HCV replication in Huh 7.5 cells inhibited autophagy by BECN1 degradation by CMA [15]. BECN1 loss impairs autophagosome–lysosome fusion, leading to the accumulation of MVBs. CMA-associated BECN1 degradation in HCV-infected cells inhibits endocytosis and degradation of epidermal growth factor receptor (EGFR). We demonstrated that CMA activation compensates for impaired autophagy due to HCV-induced microbial stress [15]. Since CMA cannot degrade unfolded proteins, protein aggregates, and non-protein cargoes such as lipids and nucleic acids, we examined whether exosome release is a potential autophagy compensatory mechanism for virus and cell survival under excessive HCV-induced microbial stress.

This study provides evidence suggesting that the early stage of persistent HCV infection induces autophagy and MVB degradation. TSPAN-CD63 bridges the degradation of MVBs through autophagic endosomal fusion in the HCV model. We found that persistent HCV infection blocks the degradation of MVBs and promotes exosome release. Increased CD63-GFP expression was observed in the late-stage of persistent HCV infection, suggesting MVBs degradation was impaired. All mammalian cells release EVs, whose 50–100 nm sized lipid bilayer can contain aggregated proteins and RNA. A growing body of evidence suggests that cells infected with enveloped or non-enveloped viruses release EVs [28]. These vesicles carry some viral proteins, viral RNAs, and viral genetic materials. The EVs isolated from HCV culture were characterized by TEM and NTA. We showed a time-dependent release of EVs in HCV-infected cell culture. TEM pictures revealed that exosomes were approximately 100–140 nm in size. The EVs isolated from the persistently infected HCV culture are characteristic of exosomes, as they express CD63 and CD9. As expected, we found that EV secretion is increased during persistent HCV infection. The peak of EV release matches with autophagy impairment at the late stage of ongoing HCV infection.

We aimed to understand the importance of EV release in viral survival by blocking EV release. Here, we show that some inhibitors suppressed EVs release in HCV culture. Blocking EVs and exosome release suppressed HCV replication in infected and replicon cell culture models. Furthermore, our data show EV release is favorable for sustaining virus replication during persistent infection. Our study investigated a potential virus–cell survival program under extreme microbial stress during chronic HCV infection. Interferons (IFNs) play a vital role in antiviral defense, mediated by innate immunity. There are three types of IFNs. IFN family comprises type I, Type II and Type III. There are differences in the mechanisms of expression and antiviral potency of antiviral cytokines in HCV infection. While type I IFNs trigger strong antiviral response in some viruses, but are not very effective in clearing chronic HCV infection, type III IFNs have a prominent effect on HCV clearance [42,43,44,45]. We speculate that TLR3-mediated recognition of double-stranded RNA (dsRNA) formed during extracellular vesicle accumulation activate type III IFN production. Future investigations will examine whether the activation of the Toll-like receptor (TLR) family, Retinoic Acid-Inducible Gene-1 (RIG-1) or Melanoma Differentiation-Associated Protein 5 (MDA5), is involved in type III IFN induction when extracellular vesicle is inhibited.

Extracellular vesicles are also involved in the pathogenesis of chronic HCV infection. During the last few years, many researchers have reported that exosomes released by HCV-infected cells play multiple roles in human liver disease progression [46]. Those studies claim that exosomes produced from HCV-infected cells carry small RNA and protein cargoes that can transfer information for cell-to-cell communication. Some studies demonstrated that exosomes contain HCV RNA and virus particles that can initiate new infections [47,48,49]. Exosomes released by HCV-infected cells are involved in the modulation of dendritic cell function and inhibit innate immune response, leading to immune escape [50,51]. Another report suggested that exosomes produced during HCV infection could hamper adaptive immune response and T cell function, which contribute to the development of chronic HCV infection [52,53]. A few publications showed that exosomes produced in HCV-infected cells could activate hepatic stellate cells implicated in the pathogenesis of hepatic fibrosis [54,55,56,57,58]. Our results indicate that extracellular vesicle release is an adaptive cellular response to chronic HCV infection. We propose that inhibiting EVs release can be explored as a potential therapeutic strategy to treat chronic HCV infection, as well as other positive-strand RNA viruses. Furthermore, we propose that the liver-derived extracellular vesicles can be used as a marker for monitoring hepatic stress response and liver disease progression during chronic HCV infection.

## Data Availability

All data presented within this study are available within the manuscript or the Supplementary Materials.

## Supplementary Materials

The following are available online at https://www.mdpi.com/article/10.3390/cells10050984/s1. Figure S1: MTT proliferation/viability assay for Torin1, Hydroxychloroquine, and GW4869. Figure S2: MTT proliferation/viability assay of drugs used to inhibit release of extracellular vesicles title, Figure S3: Huh 7.5 cell lines were infected with HCV GFP virus at MOI 0.01 and they were treated with exosome inhibitor drugs for 72 h at the day 9th of infection.
