# Supplementary material for: Extracellular Vesicle Release Promotes Viral Replication during Persistent HCV Infection
Source: Cells. 2021 Apr 22;10(5):984. doi: 10.3390/cells10050984 (PMC8146326; doi:10.3390/cells10050984)
Supplement: Supplementary file 1 [file cells-10-00984-s001.zip › Supplementary files/Supplementary Figure Legends.docx]

Supplementary Figure Legends

Figure S1: Huh 7.5 cell lines were used for 72 hours in the MTT assay of Torin 1, HCQ, and GW4869. The line graphs represent the logarithmic transformation of the normalized viability ratio of cells. Error bars represent the standard deviation of 3 different measurements.

Figure S2: MTT assay of drugs used to inhibit the release of extracellular vesicles. Huh, 7.5 cells were used in the 72-hour MTT assay of imipramine. D-pantethine, Y27632, calpeptin, manumycin A, and cytochalasin D. The line graphs represent the logarithmic transformation of normalized viability ratio of cells. Error bars represent the standard deviation of 3 different measurements.

Figure S3: Huh 7.5 cell lines were infected with HCV GFP virus at MOI 0.01 and they were treated with exosome inhibitor drugs for 72 hours at the day 9th of infection. Virus replication was evaluated with GPF expression in fluorescent microscopy (A) and Flow cytometry analysis (B). Drugs were used as following doses; Imipramine 10 µM, D-Pantethine 100 µM, Y27632 10 µM, calpeptin 30 µM, manumycin A 2 µM, cytochalasin D 2 µM and GW4869 10 µM.
