# Supplementary figures and images for: Extracellular Vesicle Release Promotes Viral Replication during Persistent HCV Infection
Source: Cells. 2021 Apr 22;10(5):984. doi: 10.3390/cells10050984 (PMC8146326; doi:10.3390/cells10050984)

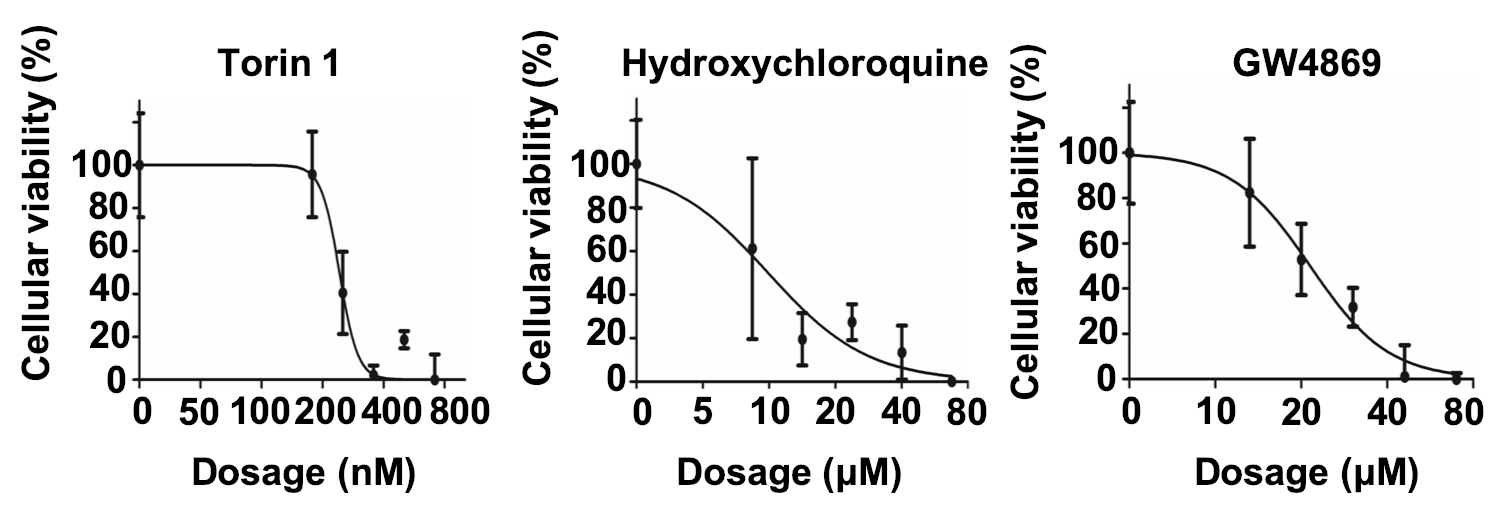

Supplement: Supplementary file 1 [file cells-10-00984-s001.zip › Supplementary files/Suppl Fig 1.tif]

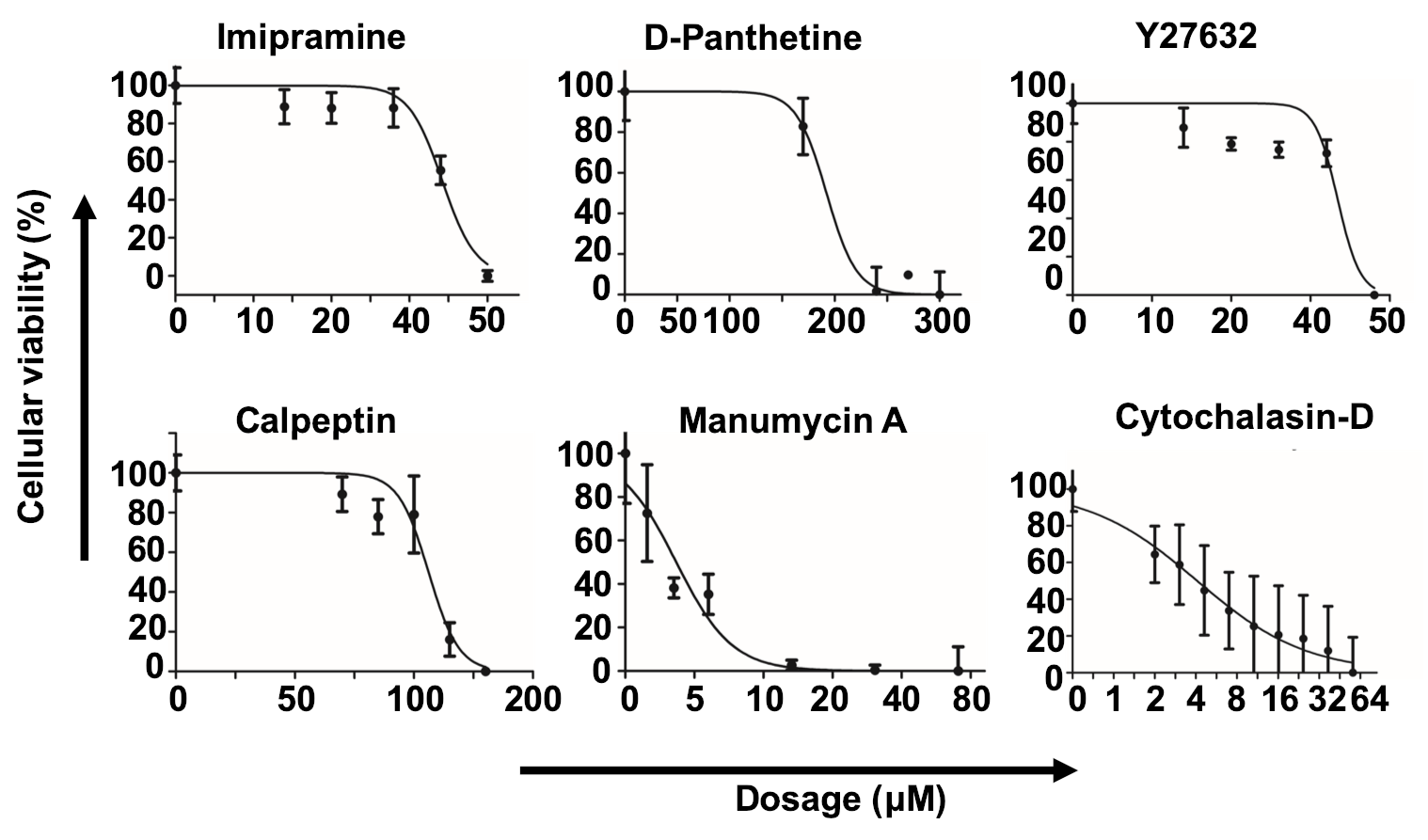

Supplement: Supplementary file 1 [file cells-10-00984-s001.zip › Supplementary files/Suppl Fig 2.tif]

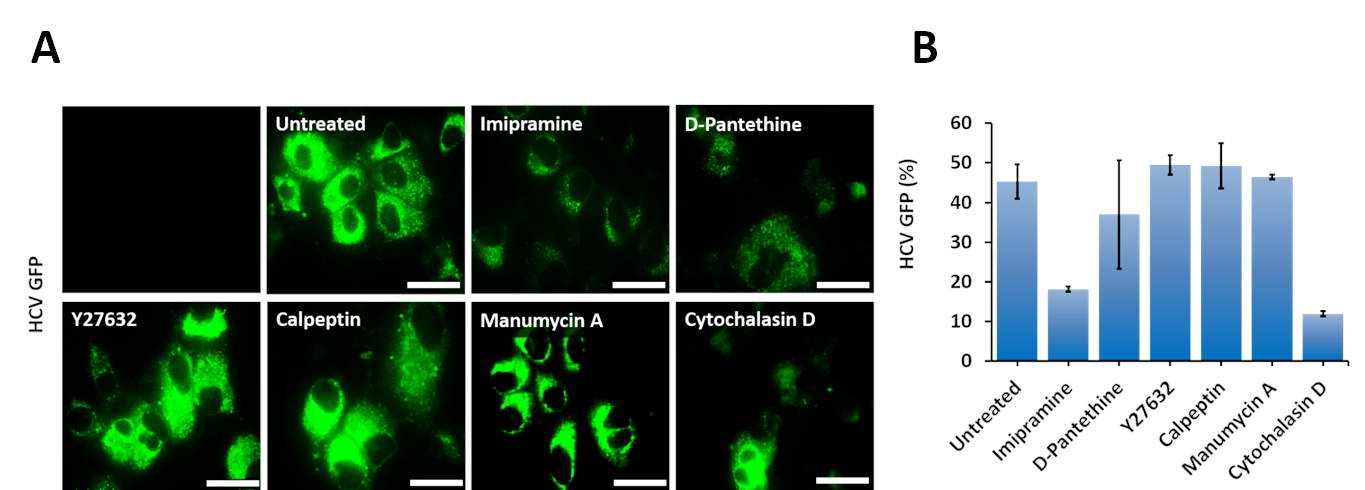

Supplement: Supplementary file 1 [file cells-10-00984-s001.zip › Supplementary files/Suppl Fig 3.tif]
